# Ventilation in American Dwellings

**Published:** 1858-10

**Authors:** 


ART. IV.— *Ventilation in American Dwellings, with a Series of Diagrams presenting Examples of Different Classes of Habitations.* By DAVID BOSWELL REID, M. D., F. R. S. E., Fellow of the Royal College of Physicians, Edinburgh; Honorary Member of the Imperial Medico-Chirurgical Society of St. Petersburgh; formerly Director of the Ventilation at the Houses of Parliament, London, etc., etc. To which is added an Introductory Outline of the Progress of Improvement in Ventilation. By ELISHA HARRIS, M. D., Late Physician in Chief of the N. Y. Quarantine Hospitals; Fellow of the College of Physicians and Surgeons of the State of N. Y.; Fellow of the N. Y. Academy of Medicine; Member of the American Medical Association, etc., etc. New York: WILEY & HALSTED, 351 Broadway. 1858.

One of the most important, and perhaps the most important subject which can engage the attention of those interested in sanitary reform, and one which is regarded with the least attention by the ordinary medical practitioner, is the subject of ventilation. Its importance and its necessity to the health of large cities, is sufficiently apparent to every one; and yet in few libraries do we find any systematic work on the subject. If there be any which treats of this subject as elaborately as the one before us, it has never been very generally known.

The work before us is prefaced by an "introductory outline of the progress of sanitary reform," by Dr. Harris, of New York, who has lately been physician in chief to the N. Y. Quarantine Hospitals, and whose studies and experience have admirably fitted him for a review of the subject and of the labors of Dr. Reid. This introductory sketch gives an interesting account of the subject of ventilation in the times of Celsus, Hippocrates and Pliny; the memorable London plague and the absolute redemption of the city by the immense conflagration on the following year; the efforts to improve the atmosphere of the British Houses of Parliament, by Sir Christopher Wren and Desaguliers; and various pestilences which have manifestly arisen from defective supplies of air. The improvements instituted by our own Dr. Franklin, by Sutton, Hales, Howard, Romford, and numerous distinguished men of their age, are passed in rapid review, showing that though the subject has always been much neglected by the people at large, it has excited the interest and called forth the earnest efforts of some of the most distinguished names which are handed down to us.

The remainder of the introductory article is rather a review of some of the points of Dr. Reid's work, and we regret that our space will not permit us to make copious extracts from it, as our notice would then do much better justice to the work. The enthusiasm of Dr. Reid led him to construct a model lecture room and practical class room, which he made to illustrate his views on the subject of ventilation, we extract from the remarks of Dr. Harris, a brief description of the building:

"The principal part of the roof was supported by fourteen pillars, which, being arranged in two rows, formed a great central colonade. Each of these pillars being hollow, constituted a fire and ventilating flue that could command four or more furnaces at its base; while the upper portion of each was perforated with a series of aparatus provided with valves, which afforded a ventilating power at any required level.

"Below the floor, flues traversed the ground in every direction, the action of many of which could be combined, whenever increased local ventilation was desired. Larger shafts or chimneys with a cupola, a steam engine, a forge, with metallic furnaces, and ventilated sand baths were added to all the peculiar apertures used in chemical experiments, and to the experimental tables provided for students.

"The lecture table was forty feet long; it was furnished with furnaces at the centre, and in the wall at each end. Steam, gas, a red heat, and ventilating power with descending currents could be secured whenever they were wanted. Behind the lecturer, on a more elevated platform, another range of furnaces and apparatus, forty feet in length, was also provided, which illustrated all the principal operations in the chemical arts, and gave great power and resources in chemical investigations. These works contributed materially to the greatly increased attention that was soon afterwards paid to ventilation and sanitary improvement."

The above is the description of a model lecture room indeed, and those who can look back to their sufferings in the crowded lecture rooms of some of our large schools, will be able to appreciate the difference such an apartment would have made in their health and comfort.

We have been thus full in noticing the introductory remarks of Dr. Harris, because it gives a key to the contents and design of the work. A close analytical or critical review, we have not the space to make; and in fact it is a work so succinct and practical in its details, that it would be impossible to do so, without repeating most of its facts and doctrines. In brief, the author goes into the subject of ventilation in all its bearings; every condition which can possibly bear upon the quality or quantity of air "necessary for different individuals in any appartment;" the arrangement of windows, doors, fireplaces, etc., in every variety of habitation; all being clearly illustrated by diagrams.

We hope that this volume will be received with the favor it deserves, both by the unprofessional as well as the professional reader. Though of great interest to us as guardians of the public health, it will be as useful to those out of our ranks, and was intended, indeed, for the general reader. We are confident that the bills of mortality, especially in our large cities and among the lower classes, would be much smaller if its precepts were more generally diffused and carried out.

It is issued in handsome style in an octavo volume, bound in muslin, by Wiley & Halsted, New York, and will be mailed to any address, prepaid, on receipt of its price ($2) in current funds or postage stamps.

